# PNPLA3—A Potential Therapeutic Target for Personalized Treatment of Chronic Liver Disease

**DOI:** 10.3389/fmed.2019.00304

**Published:** 2019-12-17

**Authors:** Xiaocheng Charlie Dong

**Affiliations:** Center for Diabetes and Metabolic Diseases, Department of Biochemistry and Molecular Biology, Indiana University School of Medicine, Indianapolis, IN, United States

**Keywords:** PNPLA3, rs738409, nonalcoholic steatohepatitis, alcoholic liver disease, fibrosis, cirrhosis, hepatocellular carcinoma

## Abstract

Patatin-like phospholipase domain-containing protein 3 (PNPLA3) is a lipid droplet-associated protein that has been shown to have hydrolase activity toward triglycerides and retinyl esters. The first evidence of PNPLA3 being associated with fatty liver disease was revealed by a genome-wide association study (GWAS) of Hispanic, African American, and European American individuals in the Dallas Heart Study back in 2008. Since then, numerous GWAS reports have shown that PNPLA3 rs738409[G] (148M) variant is associated with hepatic triglyceride accumulation (steatosis), inflammation, fibrosis, cirrhosis, and even hepatocellular carcinoma regardless of etiologies including alcohol- or obesity-related and others. The frequency of PNPLA3(148M) variant ranges from 17% in African Americans, 23% in European Americans, to 49% in Hispanics in the Dallas Heart Study. Due to high prevalence of obesity and alcohol consumption in modern societies, the PNPLA3(148M) gene variant and environment interaction poses a serious concern for public health, especially chronic liver diseases including alcohol-related liver disease (ALD) and nonalcoholic fatty liver disease (NAFLD). Therefore, PNPLA3(148M) variant is a potential therapeutic target for chronic liver disease in the rs738409 allele carriers. Currently, there is no approved drug specifically targeting the PNPLA3(148M) variant yet. With additional mechanistic studies, novel therapeutic strategies are expected to be developed for the treatment of the PNPLA3(148M) variant-associated chronic liver diseases in the near future.

Alcoholic and non-alcoholic fatty liver diseases (ALD and NAFLD) have become serious public health burdens in the modern societies (1). ALD and NAFLD are chronic liver disorders that begin with hepatic triglyceride accumulation (steatosis) and progress to hepatic inflammation and fibrosis, cirrhosis and even liver cancer (2, 3). The causes of these liver diseases are multifactorial, including genetic, and environmental factors. Excess alcohol consumption, over nutrition, and physical inactivity are significant environmental risk factors (4, 5). It is believed that hepatic steatosis sets a stage for elevated susceptibility to acute and chronic inflammation in the liver. Multiple cytokines and chemokines including transforming growth factor-β (TGF-β) secreted from inflammatory immune cells trigger an activation of hepatic stellate cells (HSCs) and subsequently hepatic fibrogenesis (6).

In addition to those environmental factors, numerous genetic variants have been shown to be associated with ALD and NAFLD, including patatin-like phospholipase domain-containing protein 3 (PNPLA3), transmembrane 6 superfamily member 2 (TM6SF2), glucokinase regulator (GCKR), membrane bound O-acyltransferase domain-containing 7 (MBOAT7), and hydroxysteriod 17-beta dehydrogenase 13 (HSD17B13) (7, 8). TM6SF2 is involved in the VLDL secretion (9–15). The rs58542926 C>T variant of TM6SF2 decreases the VLDL secretion and increases hepatic triglycerides (16–25). GCKR regulates the glucokinase activity in the liver (26). The rs780094 A>G and rs1260326 C>T variants of GCKR lead to the loss of control of hepatic glucose influx and therefore increase hepatic lipogenesis (27–38). MBOAT7 catalyzes the acyl chain remodeling of phosphatidylinositol and decreases free arachidonic acid levels (39, 40). The rs641738 C>T variant of MBOAT7 increases arachidonic acid levels and hepatic inflammation (41–54). HSD17B13 has been shown to have retinol dehydrogenase activity (55). The rs72613567:TA variant of HSD17B13 is associated with increased steatosis and decreased inflammation and fibrosis (56–64). PNPLA3 has drawn a remarkable attention in the liver field since the first genome-wide association study (GWAS) revealed that a single nucleotide polymorphism (SNP) in the human PNPLA3 gene—rs738409[G] (148M) is the only non-synonymous sequence variant significantly associated with hepatic fat content in the Dallas Heart Study cohort (65). Multiple genetic studies have since validated the association of PNPLA3(148M) with a broad spectrum of liver diseases ranging from ALD and NAFLD, non-alcoholic steatohepatitis (NASH), fibrosis, cirrhosis, and hepatocellular carcinoma (HCC) (33, 66–117). However, the underlying pathogenic mechanisms remain elusive. This review aims to briefly summarize the PNPLA3 biology, clinical implications, and therapeutic development strategies.

## PNPLA3 Gene Function

**Figure 1:**
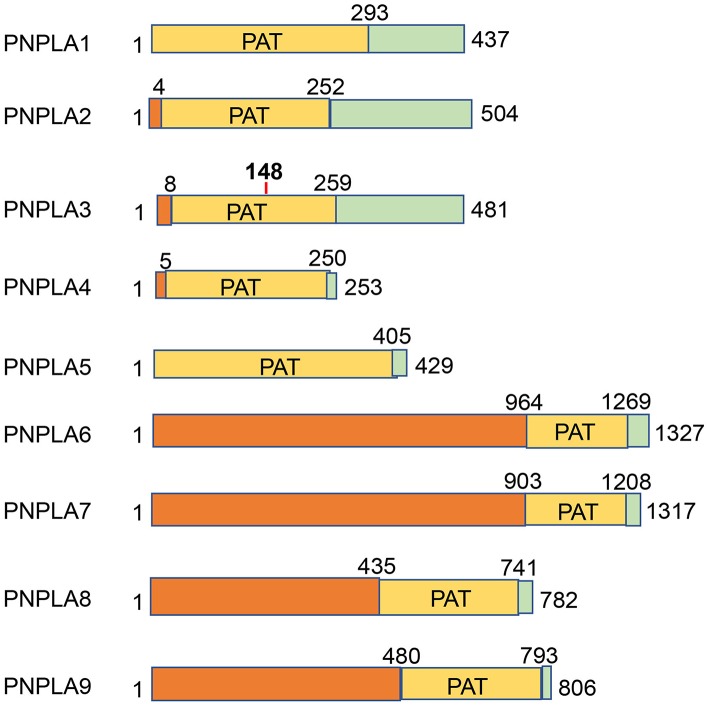
PNPLA family members. Nine PNPLA family members are depicted by the size and localization of the conserved patatin (PAT) domains.

PNPLA3 has multiple names in the literature including adiponutrin (ADPN), calcium-independent phospholipase A2-epsilon (IPLA2epsilon, and chromosome 22 open reading frame 20 (C22orf20). In 2001, PNPLA3 was initially cloned from mouse 3T3 preadipocytes as a feeding-inducible gene, therefore named adiponutrin (118). In 2004, PNPLA3 was rediscovered as IPLA2epsilon by nucleotide sequence similarity search (119). In 2006, human patatin-like phospholipases including adiponutrin were grouped to the PNPLA family (120), which has 9 members (PNPLA1-9). The common feature of the PNPLA family members is the patatin-like phospholipase domain (Figure 1). Protein sequence alignments show that the overall sequence conservation is low except a few conserved regions including the glycine-rich region and the aspartate-glycine residues of the catalytic site (120).

## PNPLA3 Gene Characterization

**Figure 2:**
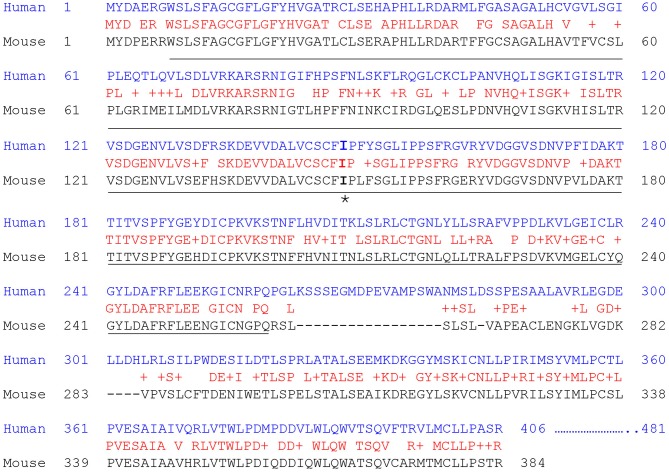
Human and mouse Pnpla3 protein sequence alignments. The protein sequences were aligned using the NCBI BLAST program. The identical residues are in red. The PAT domain is underlined. The 148I residue is marked by asterisk.

Human PNPLA3 gene is localized on chromosome 22 (22q13.31). It has 9 exons that encode a 481-amino acid protein. In contrast, mouse Pnpla3 (384 amino acids) is much smaller than human PNPLA3 protein (Figure 2), as both proteins share high homology in the N-terminal half of the amino acid sequences. But the mouse Pnpla3 lacks the middle 17 residues and the C-terminal 75 residues in the human PNPLA3 protein. Therefore, it should be cautioned when implying the mouse Pnpla3 function to human PNPLA3. Another major difference between mouse and human PNPLA3 genes is the tissue-wise gene expression profiles. The human PNPLA3 gene is expressed highly in the liver and moderately in the adipose tissue, brain, kidney, and skin (120, 121); however, the mouse Pnpla3 gene is expressed at very high levels in both white and brown adipose tissues but at low levels in other tissues (118, 122). PNPLA3 is regulated by carbohydrate-response element binding protein (ChREBP) and sterol regulatory element binding protein 1c (SREBP1c) in mouse and human hepatocytes (123–125). Surprisingly, Pnpla3 gene knockout mice have normal levels of plasma and hepatic triglyceride contents and they do not develop fatty liver disease (126, 127). Interestingly, human PNPLA3(148M) transgenic mice develop hepatic steatosis on chow or high-sucrose diet (128). Pnpla3(148M) knockin mice also develop hepatic steatosis on the high-sucrose diet (129, 130) and hepatic inflammation and fibrosis on a NASH diet (131).

## PNPLA3 Enzymatic Activities

PNPLA3 has been shown to possess triacylglycerol lipase and acylglycerol transacylase activities using recombinant human PNPLA3 protein purified from Sf9 insect cells and triolein and mono-olein as substrates, respectively (119). However, when Huang et al. used similar recombinant human PNPLA3 protein from Sf9 cells to analyze lipase and transacylase activities, they only detected the lipase activity against major glycerolipids including triacylglyceride, diacylglyceride, and monoacylglyceride but not transacylase activity (132). In another study, human PNPLA3 was overexpressed and purified from HEK293 cells and showed to have a lipase activity on 1,2-o-dilauryl-rac-glycerol-3-glutaric acid-(6'-methylresorufin) ester (122). Mutation of the active-site serine within the Ser^47^-Asp^166^ catalytic dyad motif abolished the lipase activity; however, overexpression of human PNPLA3 in HEK293 cells did not decrease the cellular triglyceride levels (122). The recombinant human PNPLA3(148M) mutant from Sf9 cells was shown to lose the triglyceride hydrolase activity using triolein as substrate (133). Human wildtype PNPLA3 but not the 148M mutant recombinant protein from yeast cells also showed triglyceride hydrolase activity (134). In addition, wildtype recombinant human PNPLA3 protein purified from yeast cells also showed retinyl esterase activity using retinyl-palmitate as substrate whereas the 148M mutant protein had diminished activity (135). Retinoic acids (all-trans) have been shown to activate retinoic acid receptor (RAR) and retinoid X receptor (RXR) and subsequently downregulate fibrotic genes in HSCs (136–138). PNPLA3(148M) mutant causes an decrease in retinol levels and downregulation of RAR/RXR target genes in the LX-2 hepatic stellate cell line (139).

## PNPLA3 in Lipid Droplet Homeostasis

**Figure 3:**
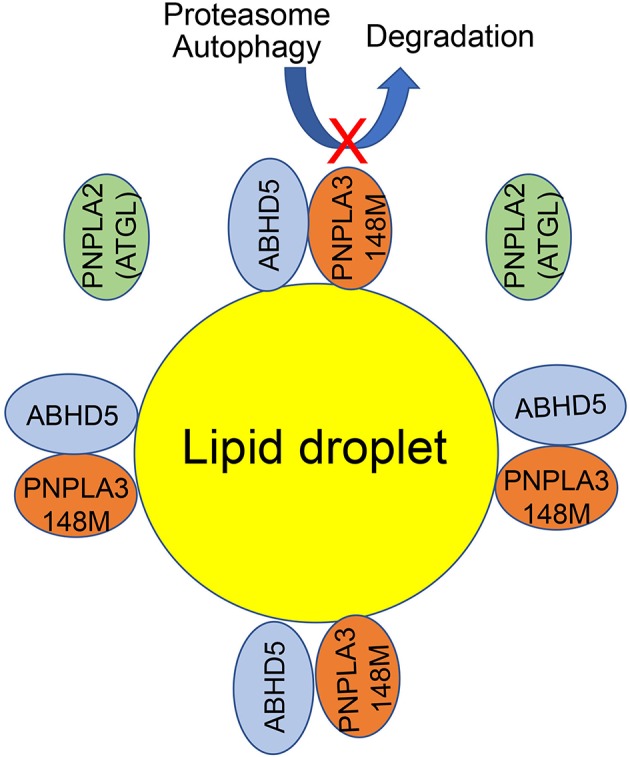
A working model for the PNPLA3 function on lipid droplet. ATGL and ABHD5 normally interact to promote triglyceride breakdown from lipid droplets. The 148M mutation impairs the turnover of PNPLA3 protein by ubiquitin or autophagy mediated degradation. When PNPLA3(148M) variant proteins accumulate on lipid droplets, PNPLA3(148M) competes with ATGL for the interaction with ABHD5. As a result, the ATGL activity is reduced and lipid droplets are accumulated.

PNPLA3 is mostly bound to lipid droplets in mammalian cells (133, 140–142), but how this protein functions on lipid droplet remains elusive (Figure 3). Several lines of evidence suggest that PNPLA3(148M) abnormally accumulating on lipid droplets links to the impairment of lipid droplet metabolism. Wildtype PNPLA3 turns over according to fasting/feeding cycles; however, the 148M mutant PNPLA3 is resistant to ubiquitin- or autophagy-mediated protein degradation (129, 143, 144). Excess PNPLA3 on the lipid droplets seems to impair the activity of PNPLA2, also called adipose triglyceride lipase (ATGL), likely through competing with the ATGL activator —comparative gene identification 58 (CGI-58) or officially abhydrolase domain containing 5 (ABHD5) (140, 142, 145). Some data suggest that PNPLA3(148M) tends to interact with CGI-58 more strongly than the wildtype counterpart does (145). CGI-58 is also required for the targeting of PNPLA3 to lipid droplet since PNPLA3 cannot localize onto lipid droplet in the CGI-58 knockout liver cells (140).

## PNPLA3 in Hepatic Fibrosis

In addition to hepatocytes, human PNPLA3 gene is also abundantly expressed in HSCs (121, 139). PNPLA3 can be induced by TGF-β but not platelet-derived growth factor (PDGF) in human HSCs (146). The same report also shows that overexpression of the wildtype PNPLA3 but not the PNPLA3(148M) mutant reduces the intracellular retinyl esters in HSCs. Interestingly, after incubation with retinol and palmitate, wildtype, but not mutant PNPLA3 decreases the secretion of matrix metallopeptidase 2 (MMP2), tissue inhibitor of metalloproteinase 1 (TIMP1), and TIMP2 from HSCs (146). Another report shows that the PNPLA3 gene expression is induced during the primary human HSC activation and knockdown of PNPLA3 by siRNA attenuates the HSC activation (139). Human HSCs with the PNPLA3(148M) variant have higher expression of inflammatory cytokines and chemokines including granulocyte-macrophage colony-stimulating factor (GM-CSF), chemokine (C-X-C motif) ligand 8 (CXCL8), and TGF-β. Overexpression of the PNPLA3(148M) variant enhances the HSC proliferation and chemotaxis (139). In contrast to the previous report regarding the retinyl palmitate lipase activity of PNPLA3 (135), Bruschi et al. have found that total retinol content and RXR and RAR signaling are both lower in the PNPLA3(148M) mutant HSCs than that in the PNPLA3 wildtype HSCs (139). Further signaling analysis has revealed that c-Jun N-terminal kinase (JNK) is highly activated in the PNPLA3(148M) HSCs. As a consequence, peroxisome proliferator-activated receptor gamma (PPARγ), a key HSC quiescence regulator, is inhibited, whereas activator protein 1 (AP-1), a proinflammatory transcription factor, is activated (139). Collectively, these dysregulations contribute to the fibrogenic phenotype in the PNPLA3(148M) HSCs. The inhibition of PPARγ in the PNPLA3(148M) HSCs also negatively affects the liver X receptor alpha (LXRα) activity. As a result, cholesterol is accumulated in those mutant HSCs, and this also contributes to the inflammation and fibrogenesis in the PNPLA3(148M) HSCs (147).

## PNPLA3 Gene Polymorphism and Chronic Liver Disease

**Figure 4:**
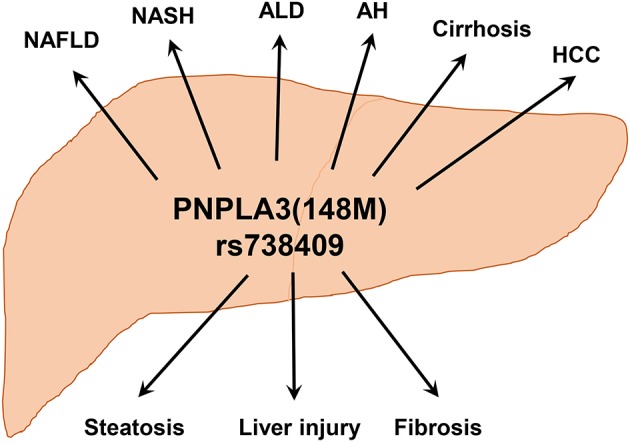
PNPLA3(148M) is associated with a wide-spectrum of chronic liver diseases. Hepatic accumulation of PNPLA3(148M) protein leads to triglyceride accumulation, liver injury, and fibrosis. With different etiologies, this may lead to the development of various liver disorders including NAFLD, NASH, ALD, alcoholic hepatitis (AH), cirrhosis, and HCC.

Alcoholic and non-alcoholic liver diseases often begin with simple steatosis and progress to hepatitis, fibrosis/cirrhosis, and even liver cancer. Both environmental and genetic factors contribute to the development of these chronic liver diseases. Among the well documented genes, PNPLA3 has the broad impact on ALD and NAFLD. The involvement of PNPLA3 variant rs738409 (148 M) in the broad spectrum of chronic liver disease has been shown by numerous GWAS (see Table 1). In 2008, Romeo et al. identified a strong association between the PNPLA3(148M) variant and hepatic fat concentration in a GWAS on Hispanic, African American, and European American individuals (65). The 148M variant frequencies are concordant with the prevalence of NAFLD in these three ancestry groups, and their allele frequencies are: Hispanics (0.49), European Americans (0.23), and African Americans (0.17). Since then, multiple GWASs have reported a strong association of PNPLA3(148M) variant with both ALD and NAFLD (Table 1 and Figure 4). Several studies have documented a strong association of the 148 M variant with liver cirrhosis (42, 76, 154, 158–160). A number of reports have also shown that the 148M variant is also associated with higher risk for HCC (77, 85, 92, 93, 106, 108, 153, 160–169). In addition, the PNPLA3 variant rs738409 could lead to differential gene regulation via microRNAs. An in silico analysis has identified hsa-miR-769-3p and hsa-miR-516a-3p as potential microRNAs targeting the 3' UTR of the human PNPLA3 mRNA (170). Experimental validations are needed to demonstrate their functional relevance to the PNPLA3(148M) variant.

**Table 1:** Human PNPLA3 genetic association studies in liver diseases.

**PNPLA3 SNP**	**Study population**	**Associated phenotype and significance**	**References**
rs738409[G]	Hispanics, African Americans, European Americans, *N* = 9,229	Positive association with hepatic fat content (*P* = 5.9 × 10^−10^), serum ALT (*P* = 1.3 × 10^−5^ in Hispanics)	(65)
rs2281135[A], rs738409[G]	Europeans, *N* = 12,419	Positive association with ALT (*P* = 8.4 × 10^−16^, *P* = 3.7 × 10^−10^)	(110)
rs738409[G]	West-Eurasian populations, *N* = 23,274	Negative association with total cholesterol (*P* = 8.87 × 10^−7^), non-HDL cholesterol (*P* = 2.27 × 10^−6^), LDL cholesterol (*P* = 7.99 × 10^−4^)	(148)
rs738409[G]	Mestizo (mixed European and Native American ancestry), *N* = 1,221	Positive association with ALD (OR = 1.45, *P* = 8.4 × 10^−4^) and alcoholic liver cirrhosis (OR = 2.25, *P* = 1.7 × 10^−10^)	(89)
rs738409[G]	Caucasian (82.1%), African American (2.3%), Asian (5.4%), American Indian (3.2%), other (7%), *N* = 1,117	Positive association with hepatic steatosis (OR = 1.46, *P* = 0.03), portal inflammation (OR = 1.57, *P* = 2.5 × 10^−4^), lobular inflammation (OR = 1.84, *P* = 0.005), Mallory-Denk bodies (OR = 1.6, *P* = 0.015), NAFLD activity score (*P* = 0.004), hepatic fibrosis (OR = 1.5, *P* = 7.7 × 10^−6^)	(68)
rs738409[G]	Japanese, *N* = 831	Positive association with NAFLD (OR = 1.73, *P* = 9.4 × 10^−10^)	(149)
rs738409[G]	German, *N* = 1,419	Positive association with alcoholic liver cirrhosis (OR = 2.79, *P* = 1.6 × 10^−6^)	(84)
rs738409[G]	Americans and Europeans, *N* = 1,997	Positive association with NAFLD (OR = 3.26, *P* = 3.6 × 10^−43^)	(83)
rs738409[G]	European Caucasians, *N* = 537	Positive association with chronic hepatitis C related hepatic steatosis (OR = 2.55, *P* = 0.034), fibrosis (OR = 3.13, *P* = 0.002)	(94)
rs738409[G]	German, *N* = 899	Positive association with liver cirrhosis (OR = 1.56, *P* = 0.005)	(150)
rs738409[G]	European Caucasians, *N* = 658	Positive association with liver cirrhosis (OR = 2.08, *P* = 0.02)	(91)
rs738409[G]	Japanese, *N* = 1,326	Positive association with NAFLD (OR = 2.05, *P* = 6.8 × 10^−14^)	(151)
rs738409[G]	American Caucasians, African Americans, Mexican Americans, *N* = 4,804	Positive association with hepatic steatosis and high ALT (OR = 1.36, *P* = 0.01)	(152)
rs738409[G]	American Caucasians, *N* = 751	Positive association with HCC (OR = 3.21, *P* = 0.02)	(153)
rs738409[G]	European Caucasians, *N* = 2,138	Positive association with alcoholic liver cirrhosis (OR = 2.19, *P* = 1.54 × 10^−48^)	(42)
rs738409[G]	Chinese Han, *N* = 768	Positive association with NAFLD (OR = 1.52, *P* = 8.7 × 10^−4^)	(102)
rs738409[G]	Eastern European, *N* = 969	Positive association with liver fibrosis (OR = 1.65, *P* = 0.001), liver cirrhosis (OR = 1.92, *P* = 5.57 × 10^−7^)	(154)
rs738409[G]	European Caucasians, *N* = 183	Positive association with alcoholic hepatitis (OR = 1.9, *P* = 0.01)	(155)
rs738409[G]	Korean, *N* = 4,409	Positive association with NAFLD (OR = 1.54, *P* = 1.74 × 10^−15^)	(156)
rs738409[G]	Chinese Han, *N* = 1,152	Positive association with ALD (OR = 1.93, *P* = 6.25 × 10^−14^)	(115)
rs738409[G]	Europeans, *N* = 5,525	Positive association with HCC (OR = 1.67, *P* = 0.005), HCC in ALD patients (OR = 3.91, *P* = 1.14 × 10^−9^), HCC in non-fibrotic patients (OR = 2.19, *P* = 0.007)	(106)
rs738409[G]	American Caucasians, *N* = 9,677	Positive association with NAFLD (OR = 1.79, *P* = 1.7 × 10^−20^)	(157)
rs4823173[A], rs2896019[G], rs2281135[A]	Mexican Americans, *N* = 3,757	Positive association with AST (*P* = 3.44 × 10^−10^, *P* = 7.29 × 10^−9^, *P* = 8.73 × 10^−9^)	(109)

## Therapeutic Strategies for Targeting PNPLA3 for Personized Treatment of Chronic Liver Disease

As the PNPLA3(148M) variant is quite prevalent in most populations, especially among Hispanics (65), it is very significant to develop therapeutics targeting this genetic polymorphism. According to the PNPLA3(148M) biology, there are several potential ways of targeting the 148M variant. First, the PNPLA3(148M) variant can be targeted at the RNA levels by small interfering RNA (siRNA), small hairpin RNA (shRNA), or antisense RNA oligonucleotide. A recent report has shown that triantennary N-acetylgalactosamine (GalNAC_3_) conjugated antisense oligonucleotides (ASO) targeting Pnpla3 in a 148M knockin mouse model significantly reduce hepatic steatosis, inflammation, and fibrosis (131), suggesting the utility of the ASO strategy. In another report, targeting Pnpla3 in the 148M knockin mice by AAV-mediated shRNA has also showed effective reduction of hepatic triglyceride contents (143). For the translational perspective, PNPLA3(148M)-allele-specific RNAi is preferred for human patients in order to avoid affecting the PNPLA3 wildtype allele as we do not fully understand the PNPLA3 biology. With the encouraging phase III clinical trial data on proprotein convertase subtilisin/kexin type 9 (PCSK9) RNAi (171), targeting the PNPLA3(148M) variant by RNAi can be an attractive strategy. Second, PNPLA3 can be targeted at the protein level. Recent data suggested that an accumulation of PNPLA3(148M) on lipid droplets is very critical for the pathogenesis of fatty liver disease (129, 130, 140, 143). Therefore, targeting PNPLA3(148M) for degradation can be a useful strategy. Recently, a proof-of-concept study using proteolysis-targeting chimera (PROTAC)-mediated degradation of Halo-tagged PNPLA3(148M) has shown a significant effect on lowering hepatic triglyceride content (143). The question will be how to degrade endogenous PNPLA3(148M) protein in a variant-specific manner. To date, there are no effective ways to specifically target the PNPLA3(148M) mutant protein. However, targeting PNPLA3 may work from another angle —an interaction between PNPLA3 and CGI-58, as the interaction can be regulated by fatty acids or synthetic CGI-58 ligands (145). Taken together, targeting PNPLA3(148M) has been increasingly appreciated for therapeutic development for multiple chronic liver diseases including ALD and NASH.

In summary, PNPLA3 is an enigmatic protein that has broad implications in metabolic liver diseases from simple steatosis to cirrhosis and liver cancer. Better understanding the biological function of PNPLA3 in lipid droplet metabolism should facilitate the therapeutic development. Targeting the PNPLA3(148M) variant is expected to be an excellent example of the modern personalized medicine.

## Author Contributions

XD conceived the idea, gathered the data, and wrote the manuscript.

### Conflict of Interest

The author declares that the research was conducted in the absence of any commercial or financial relationships that could be construed as a potential conflict of interest.
